# A Rare Case of Bivalvular Infective Endocarditis With Left Atrial Mural Endocarditis

**DOI:** 10.7759/cureus.62268

**Published:** 2024-06-12

**Authors:** Aakash D Rana, Jack Xu, Rupesh Manam, Brittany A Zwischenberger, Rahel Alemu

**Affiliations:** 1 Department of Medicine, Central Arkansas Veterans Healthcare System, Little Rock, USA; 2 Department of Cardiology, Novant Health, Winston-Salem, USA; 3 Department of Cardiothoracic Surgery, Duke University, Durham, USA

**Keywords:** transthoracic echocardiogram, transesophageal echocardiogram (tee), non-valvular endocarditis, mural endocarditis, mitral regurgitation, left atrial endocarditis

## Abstract

Infective endocarditis (IE) is a life-threatening cardiac infection usually associated with cardiac valves. Left atrial (LA) mural endocarditis is rarely seen and occurs in isolation or in conjunction with mitral valve endocarditis. We present a case of a 61-year-old male with no prior cardiac history who presented with melena and fevers. Blood cultures were positive for Enterococcus faecalis. Transesophageal echocardiogram (TEE) demonstrated aortic and mitral valve vegetations with several small echo densities present on the left atrial wall. These findings were further assessed with a computed tomography angiogram of the heart and cardiac magnetic resonance imaging Ti600 sequence. The patient was treated with intravenous antibiotics and underwent aortic and mitral valve replacement with resection of numerous small fungating masses on the left atrium. There are currently no formal guidelines in place for managing mural endocarditis. However, conducting a multidisciplinary evaluation by an endocarditis team could aid in achieving earlier and more precise diagnoses of the underlying condition and its complications. This approach could also ensure consistent antibiotic therapy and appropriate timing for surgical intervention.

## Introduction

Infective endocarditis (IE) is a life-threatening cardiac infection associated with high mortality rates [1]. IE may present not only as an acute, rapidly progressive infection but also as a subacute or chronic disease with low-grade fever. IE should be suspected with fever with cardiac risk factors (presence of prosthetic valve or cardiac device or history of valvular or congenital heart disease) or non-cardiac risk factors (intravenous drug use, indwelling intravenous lines, or recent dental or surgical procedures) [2]. Multimodal imaging, including echocardiography, computed tomography (CT), and magnetic resonance imaging (MRI) plays an essential role in diagnosing, managing, assessing prognosis, and monitoring the condition, making it indispensable in the comprehensive care of patients with infective endocarditis [2]. Treatment of IE is composed of microbial eradication with antibiotics and surgery to help remove infected material and drain abscesses. Delays in diagnosis and treatment may be associated with complications such as valvular regurgitation, heart failure, embolic events, and sepsis [2].

While heart valves represent the most frequent site of involvement, vegetation can also manifest in other intracardiac locations [3]. Left atrial (LA) mural endocarditis is rarely seen and occurs in isolation or in conjunction with mitral valve endocarditis [4]. A significant portion of these cases involve individuals who are either critically ill, debilitated, or immunocompromised, representing a subset with heightened susceptibility to embolic events [5]. In mural endocarditis, the common causative organisms are Staphylococcus aureus, Streptococcus, Candida, and Aspergillus species [6]. It is theorized that the jet effect of mitral regurgitation (MR) causes endothelial injury on the wall of the left atrium in the setting of bacteremia, leading to the development of left atrial mural endocarditis. We report a case of LA mural endocarditis in the setting of mitral and aortic endocarditis, which was diagnosed via multimodality cardiac imaging.

## Case presentation

**Figure 1:**
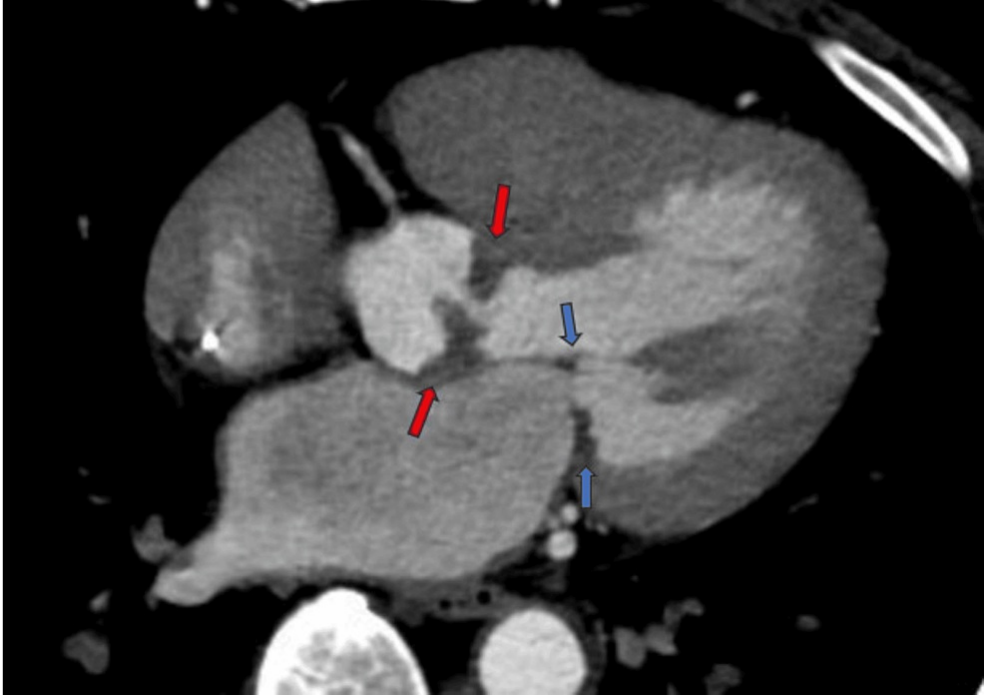
Computed tomography angiogram of the heart showing extensive aortic valve (red arrows) and mitral valve thickening (blue arrows)

**Figure 2:**
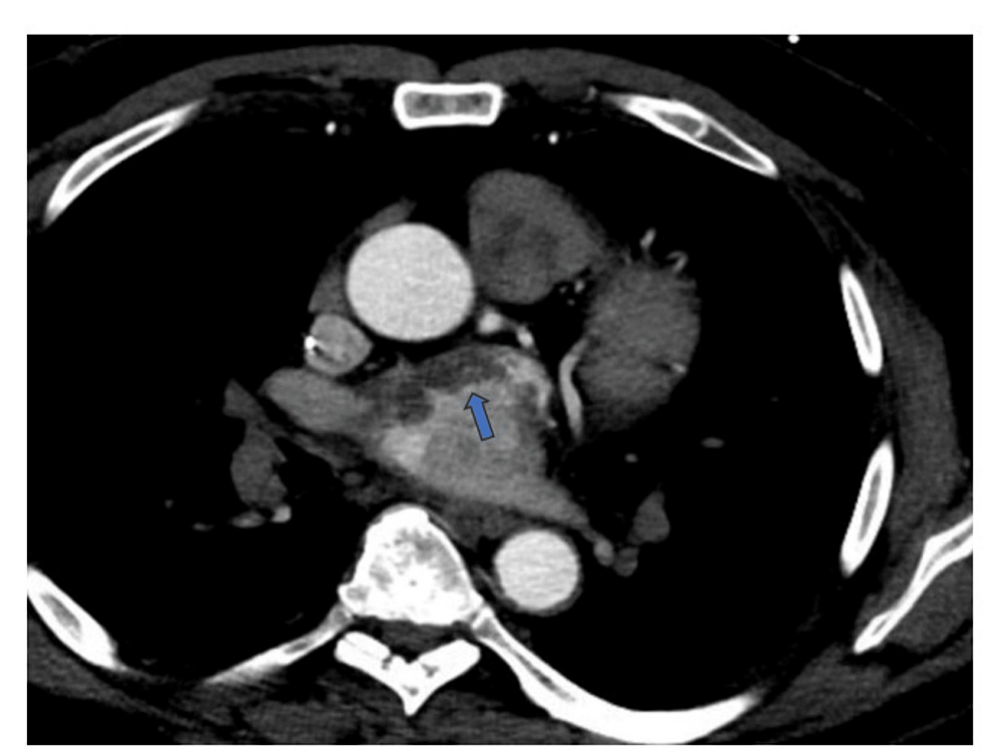
Computed tomography angiogram of the heart showing a left atrium mass

A 61-year-old male with a past medical history of hypertension and peripheral neuropathy presented to the emergency department with symptomatic anemia (hemoglobin 6.0), which was a drop from a normal level three weeks prior, and fever. Gastroenterology performed an upper endoscopy, which did not reveal any source of bleeding. The workup for bacteremia was negative. The patient stabilized and was discharged home. The patient was rehospitalized a few days later with worsening dark stools. On readmission, vital signs were stable, white blood cell count (7000/µL), hemoglobin (8 g/dL), platelets (187,000/µL), and creatine (1.3 mg/dL). Physical exam revealed a new III/VI holosystolic murmur heard best at the apex of the heart on exam. Vitals and labs were stable. A colonoscopy revealed arteriovenous malformations, which were treated with argon plasma coagulation. Repeat blood cultures on this admission grew Enterococcus faecalis. The patient was started on broad-spectrum antibiotics. Transthoracic echocardiogram (TTE) demonstrated a left ventricle ejection fraction of 55-60%, thickening of the mitral and aortic valves, moderate mitral regurgitation, and moderate mitral stenosis (MS) of 9 mmHG at a heart rate of 93 bpm in normal sinus rhythm (Video 1). Transesophageal echocardiogram (TEE) demonstrated aortic and mitral valve thickening, several echo densities adherent to the left atrial wall, and moderate mitral regurgitation. (Video 2 and Video 3). A cardiac magnetic resonance imaging (MRI) Ti600 demonstrated no enhancement of the intracardiac masses (Video 4). Computed tomography angiogram of the heart revealed extensive aortic valve thickening, mitral valve thickening, and left atrium mass (Figure 1 and Figure 2).

**Video 1:** Transthoracic echocardiogram with parasternal long-axis view showing thickened aortic valve and mitral valve TTE: transthoracic echocardiogram

**Video 2:** Transesophageal echocardiogram with mid-esophageal aortic valve long-axis view showing several echo densities adherent to the left atrial wall and thickening of the aortic valve TEE: transesophageal echocardiogram

**Video 3:** Transesophageal echocardiogram with mid-esophageal four-chamber view showing a central mitral regurgitation jet TEE: transesophageal echocardiogram

**Video 4:** Cardiac MRI with a TI 600 sequence showing a lack of late gadolinium enhancement of the aortic valve, mitral valve, and left atrial masses MRI: magnetic resonance imaging

**Figure 3:**
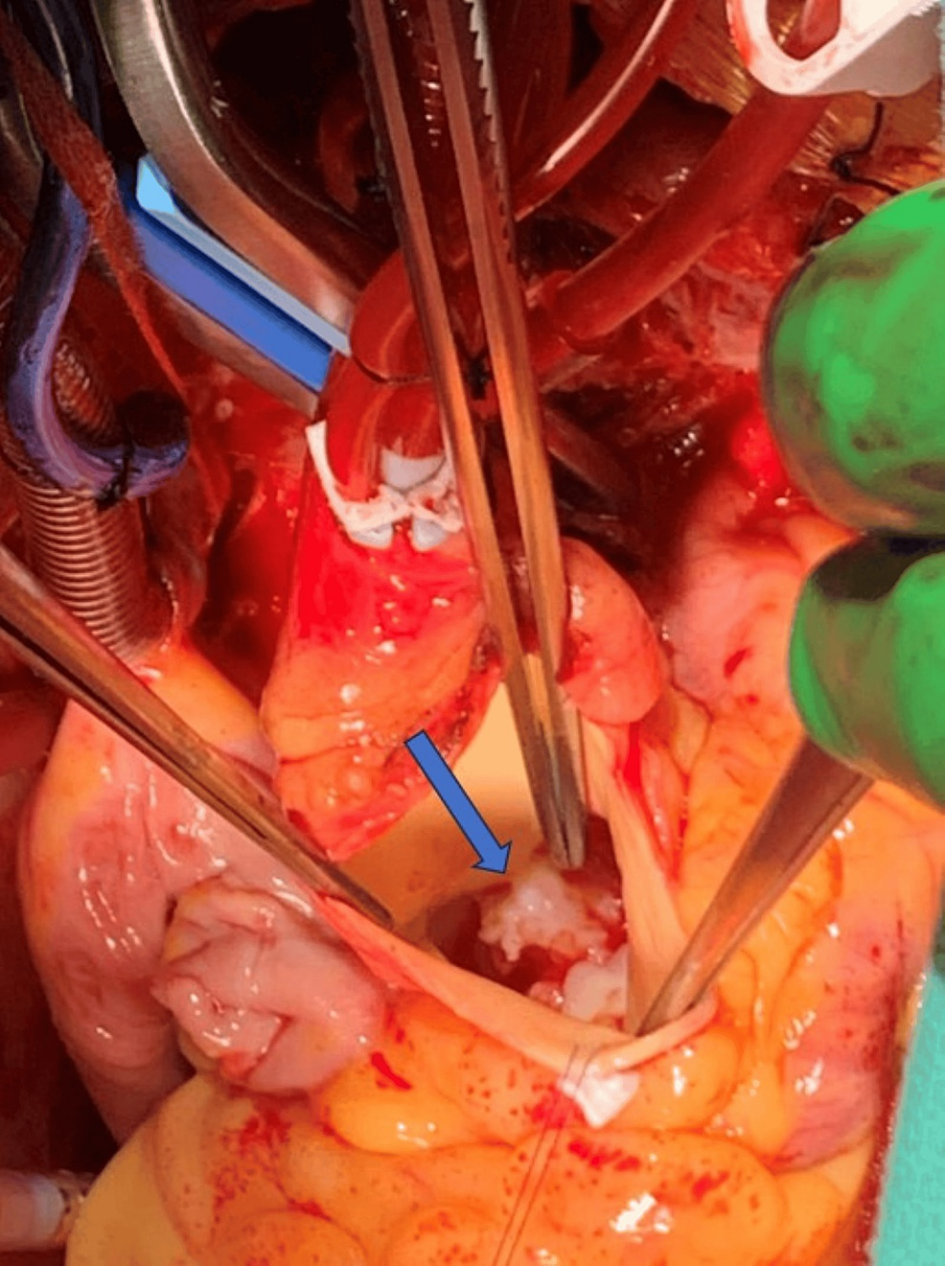
Gross image showing masses attached to the non-coronary cusp of the aortic valve, consistent with aortic valve endocarditis before valve replacement

Based on the imaging and blood cultures, a definitive diagnosis of infective endocarditis was made using the Duke criteria. Repeat blood cultures were negative on this admission. The patient was discharged home on six weeks of ceftriaxone and penicillin. The patient was referred to a cardiothoracic surgeon for further evaluation. The patient successfully underwent aortic valve replacement (St. Jude Mechanical Regent 21 mm) and mitral valve replacement (St. Jude Mechanical Valve 25 mm) (Figure 3).

**Figure 4:**
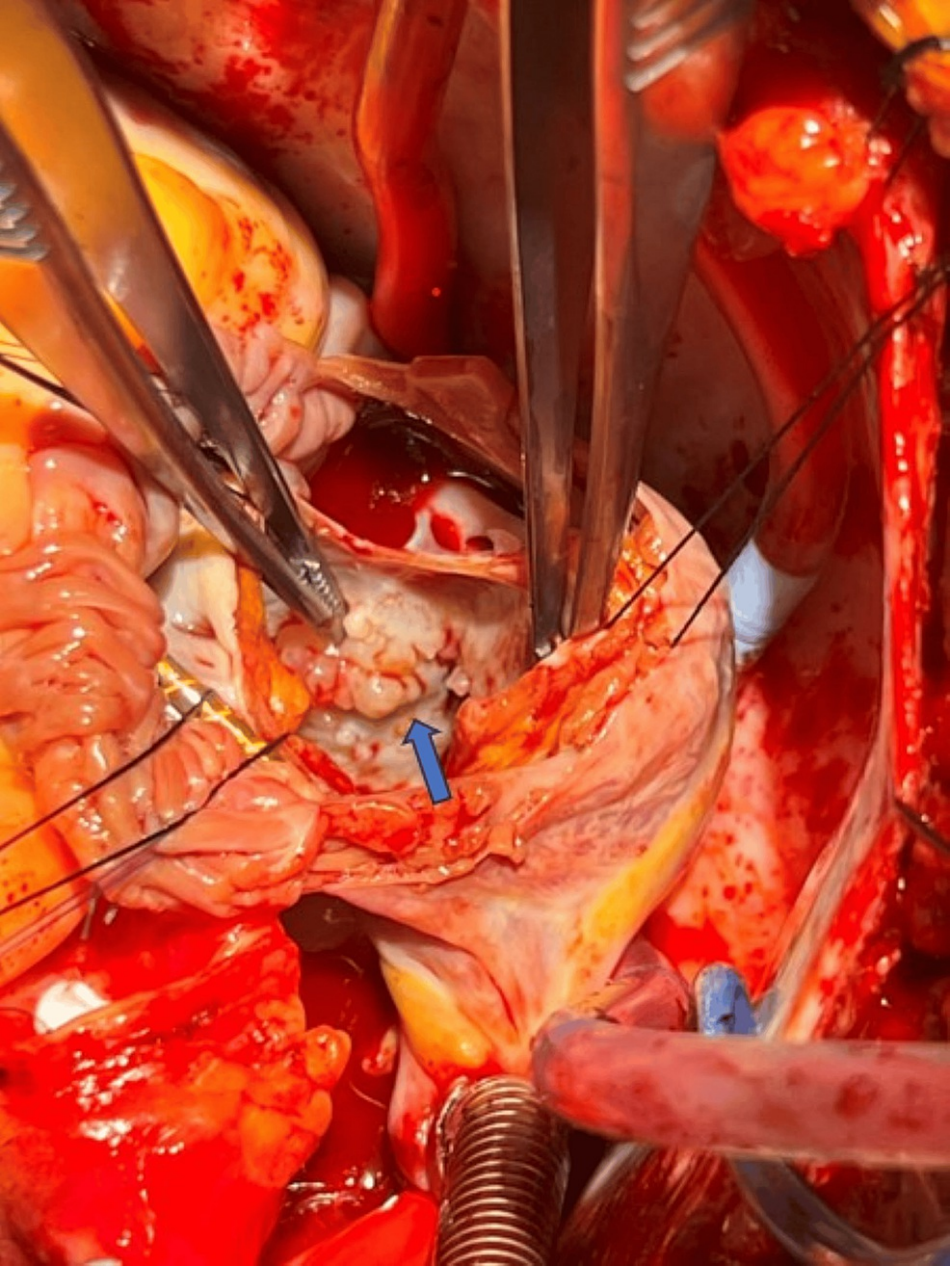
Gross image showing masses on the wall of the left atrium, consistent with left atrial mural endocarditis

During the procedure, numerous small fungating masses were noticed in the left atrium and resected during surgery (Figure 4).

Pathology of the aortic valve and left atrial mass showed Enterococcus faecalis, yet the mitral valve showed no growth of microorganisms. The patient had an unremarkable postoperative course and was discharged home.

## Discussion

Infective endocarditis is an infection of the endocardial surface of the heart caused by bacteria and fungi. Although heart valves are the most common site of involvement, vegetation can occur in other intracardiac locations such as the atrium, ventricle, and endothelium of the great vessels [7,8]. Mural endocarditis is rarely reported, and left atrial mural endocarditis is infrequently reported [9].

The emergence of infective endocarditis (IE) is linked to an underlying structural abnormality in the heart. A pre-existing valvular condition, notably mitral regurgitation (MR), is a key predisposing cardiac factor for IE. In our case, left atrial mural endocarditis was discovered in the setting of MR with no known hypercoagulable condition and in normal sinus rhythm. The etiology of mural endocarditis development occurs via two pathways: 1) regurgitant jet secondary to MR causes endothelial injury, resulting in depositions of fibrins and platelets and retrograde spread of the infection to the left atrium wall, and 2) primary mural endocarditis that occurs in chronically ill or immunodeficient patients without heart disease. Primary mural endocarditis is less common [4,10]. In our patient, vegetations were detected on the wall of the left atrium, precisely where the regurgitant jet made contact. The differential diagnosis for left atrial mass includes myxoma, thrombus, secondary cardiac tumor, and IE.

An established guideline for managing valvular endocarditis suggests that early surgical intervention should be considered when the infection is accompanied by large vegetation, major valvular complications, and peripheral embolism [11]. Timely diagnosis, antibiotics, and surgical intervention may prevent the development of complications, such as embolization, in patients with large mural vegetations [12]. No formal guidelines are established for mural endocarditis management, but a multidisciplinary heart valve team evaluation would be the best approach for these patients. In our case, the left atrial mural endocarditis was resected during the operation due to the large size and friability of the masses. No micro-organism growth was found on the cultures of the mitral valve specimen, possibly due to prior antibiotic usage [13]. Still, the cultures of the left atrial masses were positive at the time of the operation.

## Conclusions

Left atrial mural (IE) is uncommon and often occurs alongside mitral regurgitation. This is triggered by high-velocity mitral regurgitation jets causing endothelial injury, resulting in a highly thrombophilic condition that can serve as a site for infection during bacteremia. Multimodal imaging, including echocardiography, computed tomography (CT), and magnetic resonance imaging (MRI), is vital in establishing the diagnosis of left atrial IE in patients. No formal guidelines are established for mural endocarditis management, but a multidisciplinary heart valve team evaluation would be the best approach for these patients.
